# Is there a role for total neoadjuvant treatment in early-stage rectal cancer?

**DOI:** 10.1007/s00423-025-03895-2

**Published:** 2025-11-11

**Authors:** Kamil Erozkan, Metincan Erkaya, Jacob A. Miller, Ali Alipouriani, David Liska, Hermann Kessler, Scott R. Steele, Emre Gorgun

**Affiliations:** 1 Department of Colorectal Surgery, Digestive Disease and Surgery Institute, Cleveland Clinic, Cleveland, OH USA; 2 Division of Colorectal Surgery, General Surgery Department, Ege University Hospital, Izmir, Turkey; 3 Department of Radiation Oncology, Cleveland Clinic, Cleveland, OH USA; 4 Department of Colorectal Surgery , Digestive Disease and Surgery Institute, 9500 Euclid Avenue, Desk A-30, Cleveland, OH 44195 USA

**Keywords:** Early-stage rectal cancer, Stage i rectal cancer, TNT, Organ preservation

## Abstract

**Background:**

Current guidelines recommend surgical interventions for stage I rectal cancer (S1RC). Impaired functions and permanent colostomy remain undesirable outcomes, particularly in low-located stage I rectal cancer. Selective use of total neoadjuvant treatment (TNT) in S1RC may be a potential treatment option.

**Study design:**

Patients with S1RC who declined total mesorectal excision (TME) and opted for TNT between 2015 and 2023 were retrospectively reviewed. The study included two groups: (1) patients with S1RC who demonstrated a partial response following chemoradiation and chose consolidation chemotherapy, and (2) patients who underwent local excision of rectal lesions that were subsequently confirmed as S1RC but declined the recommended TME. Primary outcomes were complete response and organ preservation rates.

**Results:**

The study included sixteen S1RC patient (69% male) who underwent TNT. Eleven patients received TNT following partial response, while the remaining underwent TNT after transanal full-thickness local excision. In the first group (*n* = 11), nine patients achieved a complete clinical response. One patient with a near-complete response underwent endoscopic submucosal dissection, which revealed a tubulovillous adenoma. Another patient demonstrated a partial clinical response and subsequently underwent low anterior resection, with the final pathology showing a complete response. In patients who received TNT after local excisions, no local recurrence or distant metastasis was observed, with a median follow-up of 20 months (IQR 12). The overall complete response rate following TNT is 93.7%, and organ preservation rate of the study was 87.5%.

**Conclusion:**

Selective utilization of TNT in S1RC holds the potential to foster organ preservation, particularly for low rectal cancer. Larger prospective studies with longer follow-up and standardized treatment protocols are needed to validate these preliminary findings.

## Introduction

The management for locally advanced rectal cancer (LARC) is experiencing a paradigm shift from standard neoadjuvant therapy to total neoadjuvant therapy (TNT). TNT encompasses neoadjuvant induction or consolidation chemotherapy in addition to chemoradiation, aims to eradicate early micrometastatic disease, enhance treatment compliance, and increase complete response rates compared to adjuvant regimens [1–3]. Despite its effectiveness in achieving local tumor control, total mesorectal excision (TME) is the current standard surgical management, which is associated with considerable morbidity [4]. Patients may experience impaired functional outcomes, significantly impacting their overall quality of life, especially due to surgical intervention. Furthermore, a considerable proportion of patients undergo abdominoperineal resection (APR), which obliges patients to live with a permanent colostomy. Efforts are ongoing to prioritize organ preservation to reduce morbidity related to TME and to improve quality of life [5–7]. The increased likelihood of achieving a complete clinical response following TNT compared to standard treatment enables patients to be safely monitored non-operatively using a watch-and-wait (WW) protocol. WW has an organ-preserving potential by sparing patients from impaired functional outcomes and permanent colostomies [8–10]. However, despite achieving a complete clinical response, patients may still experience functional impairments due to the effects of radiation and chemotherapy. Ongoing research continues to investigate the long-term functional outcomes and quality of life in patients managed with WW after TNT.

On the other hand, for patients diagnosed with stage I rectal cancer, current guidelines recommend local excision techniques in selected patients. For patients with high-risk features or patients with T2 tumor, TME ± adjuvant chemotherapy is the recommended management [1, 11]. However, impaired functions and permanent colostomy remain undesirable, particularly in low-located stage I rectal cancer who underwent TME. In the paradigm shift era of rectal cancer treatments, some studies have demonstrated that patients with clinically staged T2N0 distal rectal cancer can achieve pathologic complete response following neoadjuvant chemoradiotherapy (nCRT) [12, 13]. Highlighting the importance of neoadjuvant chemotherapy, recent guidelines have endorsed a new treatment strategy of neoadjuvant therapy in highly selected patients, particularly in those cases where an abdominoperineal resection (APR) would be required [1, 11]. TNT may potentially serve as a viable treatment option for highly selected patients with stage I rectal cancer who either decline surgical intervention or are deemed ineligible candidates for surgery due to comorbidities or other conditions. The outcomes of patients with stage I rectal cancer who underwent TNT have yet to be explored.

Therefore, we aim to investigate the preliminary series of patients with stage I rectal cancer who were treated with TNT, emphasizing patient-centered decision-making. Through this investigation, we seek to contribute to the potential role of TNT in stage I rectal cancer management, with a focus on safety for oncological outcomes and treatment efficacy. We hypothesize that TNT may be considered as a potential treatment option in patients with stage I rectal cancer.

## Materials & methods

### Study design and outcomes

We conducted a retrospective analysis of patients with stage I rectal cancer who declined TME and opted for TNT at our institution between 2015 and 2023, following approval from the institutional review board. The study included two groups: (1) patients with stage I rectal cancer who demonstrated a partial response following chemoradiation and chose consolidation chemotherapy instead of TME, and (2) patients who underwent local excision of rectal lesions that were subsequently confirmed as stage I rectal cancer but declined the recommended TME based on adverse histopathological findings and opt for TNT. Although these paths are different, the main question is the same—can we manage without surgery after a complete response and keep the rectum safe from cancer? Given our small sample size, looking at these similar situations together makes our findings more applicable to real-world, patient-focused decisions.

Stage I rectal cancer is defined as stage I (cT1/2 N0/X) adenocarcinoma with a height ≤ 15 cm from the anal verge, as determined by clinical exam, flexible endoscopy and magnetic resonance imaging (MRI) findings. Thoracic, abdominal, and pelvic computed tomography (CT) scans were obtained to rule out metastases. For the resected lesions, a positive margin was defined as the presence of adenocarcinoma extending to the inked specimen margin.

The outcomes assessed in this study included tumor regrowth rate, disease-free survival time after local excision, and the duration of the WW period following TNT. While TNT has become the standard of care for LARC at our institution, its application is more selective in patients with stage I rectal cancer, based on patient-centered decision. All patients are discussed at the multidisciplinary tumor board, and final treatment decisions regarding TNT or alternative approaches are made collaboratively also discussing with the patients.

Patients receive TNT, which consists of long-course chemoradiation (50–50.4 Gy radiotherapy in 25–28 fractions with concurrent Capecitabine) followed by consolidation chemotherapy. The consolidation chemotherapy regimen typically includes 4 months of FOLFOX (fluorouracil, leucovorin, oxaliplatin; 8 cycles) or CapeOx (capecitabine, oxaliplatin; 5 cycles), tailored to the patient’s clinical profile and treatment goals. Approximately 4–8 weeks after completing TNT, patients undergo restaging through digital rectal exam (DRE), flexible sigmoidoscopy, MRI of the rectum or pelvis, and CT of the thorax, abdomen, and pelvis. cCR is determined based on post-TNT sigmoidoscopy [14] and MRI findings [15]. The absence of a palpable or residual tumor defines cCR [16]. Instead, whitening of the mucosa and inflexible rectal wall harboring the scar without additional findings may be observed during a rectal examination and sigmoidoscopy. On MRI, the diffusion sequence shows a limited signal associated with a reduction in the lesion size with residual fibrosis only [magnetic resonance tumor regression grade (mrTRG) 1]. Patients are advised to undergo active surveillance. During WW, patients who develop tumor regrowth are typically recommended to undergo TME or selectively Endoscopic submucosal dissection (ESD) in our practice. We kept DRE as a key part of our monitoring because it adds important information to endoscopy and MRI, especially for tumors lower in the rectum. DRE can detect subtle induration, mural thickening, tethering, or nodularity at the anastomotic scar that may precede endoscopic or radiologic changes, and it helps localize abnormalities seen on imaging. If DRE finds something suspicious, we quickly perform an endoscopy and MRI to check for partial response or tumor regrowth. On the other hand, a normal DRE supports a cCR when endoscopy only shows whitening or a flat scar and MRI shows mrTRG 1 fibrosis without diffusion restriction. For patients who had transanal full-thickness local excision, using DRE during WW is more difficult. Surgery changes the rectal wall, causing scarring or irregularities that make it hard to tell between normal changes and early tumor return. In these cases, small findings like hardening or thickening are hard to interpret. So, while we still did DRE for these patients, we always combined it with flexible sigmoidoscopy and pelvic MRI.

Our department’s WW protocol for rectal cancer surveillance involves digital rectal examination, carcinoembryonic antigen, and flexible sigmoidoscopy every 3 months in the first 2 years, every 6 months in years 3 and 4, and annually from years 5 to 10. MRI of the pelvis is performed every 6 months for the first 4 years and then yearly. CT of the thorax and abdomen is done every 6 months for the first 2 years and annually thereafter. Colonoscopy is scheduled 1 year after treatment followed by a 3-year interval. All patients were monitored according to a standardized surveillance protocol.

### Data collection and statistical analysis

Collected variables included patient demographics [e.g., age at diagnosis, sex, Charlson Comorbidity index [17], and body mass index (BMI)], tumor characteristics [e.g., distance to internal anal sphincter (IAS), stage, tumor location, regrowth, local recurrence and distant metastasis (DM)], TNT course, diffuse free survival time (DFS) and WW. The DFS time, defined as the interval between local excision and the most recent update, and the total WW time, defined as the interval between the TNT date and the date of tumor regrowth or the last follow-up date, were calculated in the study. Data were presented as median [interquartile range (IQR)] or frequency (percentile). Descriptive statistics were used to show our results in stage I rectal cancer following TNT. The study received approval from the Institutional Review Board.

## Results

In total, sixteen patients with rectal adenocarcinoma (69% male) who underwent TNT were included in the study. Among them, eleven patients underwent TNT after the initial diagnosis (Table 1A), while the remaining underwent TNT after transanal full-thickness local excision (Table 1B). The median age was 62.5 years (IQR 14), and the median BMI was 31.2 kg/m^2^ (IQR 8). The median for Charlson Comorbidity Index for all patients was 4 (IQR 3). Before the initiation of TNT, the median tumor size was 3.1 cm (IQR 1.3). Among patients who underwent TNT after the initial diagnosis, six had tumors abutting the IAS, while the median distance from the anal verge for the remaining five tumors was 1.4 cm (IQR 0.6). Nine patients achieved a clinical complete response following TNT, while one patient underwent endoscopic submucosal dissection due to a near-complete response, revealing a tubulovillous adenoma without dysplasia. Additionally, another patient showed partial response in final endoscopic and radiologic assessment and underwent LAR, revealing a pathologic complete response (ypT0N0). Ten patients who underwent TNT after the initial diagnosis showed no tumor regrowth, except for the patient who initially decline the surgery and opted for TNT, and exhibited a rectovaginal fistula due to the local recurrence in 6-month follow-up visit. The patient with local recurrence refused surgery after the second assessment following recurrence and opted for chemotherapy, which they are currently receiving. The median follow-up after the completion of TNT was 27 months (IQR 18), during which patients were closely monitored and exhibited no disease recurrence or mortality.

**Table 1:** Patient demographics and tumor characteristics

(A)	Age	Sex	CCI	BMI	Size (mm)	Stage	Distance to IAS (cm)	TNT Course	Tumor Board Decision	WW time (month)	Regrowth or DM
#1	74	F	5	31.1	16	T2 N0	0.7	CRT + FOLFOX	CR	19	No
#2	72	M	6	31.2	26	T2 N0	1.4	CRT + FOLFOX	CR	32	No
#3	70	M	3	26.8	40	T2 N0	abuts	CRT + FOLFOX	CR	*11*	No
#4	67	F	3	29.2	31	T2 N0	abuts	CRT + FOLFOX	CR	15	No
#5	65	M	4	31.2	25	T2 N0	abuts	CRT + FOLFOX	Near-CR ◊ ESD Tubulovillous adenoma	27	No
#6	60	M	4	26.1	45	T2 N0	abuts	CRT + FOLFOX	CR	*28*	No
#7	57	F	3	20.8	25	T2 N0	2.9	CRT + FOLFOX	CR	14	*Rectovaginal Fistula*
#8	57	F	4	36.1	31	T2 N0	abuts	CRT + FOLFOX	Partial Response	LAR ◊ T0N0 DFS: 71	No
#9	54	M	4	47.8	45	T2 N0	1.4	CRT + FOLFOX	CR	*33*	No
#10	46	M	3	27.6	34	T2 N0	0.8	CRT + FOLFOX	CR	*57*	No
#11	43	M	6	27.6	18	T2 N0	abuts	CRT + FOLFOX	CR	11	No
**(B)**	**Age**	**Sex**	**CCI**	**BMI**	**Size (mm)**	**Stage**	**Location**	**TNT Course**	**Histopathology**	**DFS**	**LR or DM**
#12	70	F	6	35.1	26	pT1 Nx	Lower Rectum	CRT + FOLFOX	IA, mucinous type *< 1 mm margin*	20	No
#13	69	M	6	35.1	25	pT1 Nx	Lower Rectum	CRT + CapOx	IMDA *negative margins* *PNI*	20	No
#14	65	M	7	41.4	n/a	pT2 Nx	Lower Rectum	CRT + CapOx	IMDA *positive margins*	32	No
#15	59	M	4	30.8	37	pT1 Nx	Lower Rectum	CRT + FOLFOX	IMDA *positive margins*,* LVI*	45	No
#16	39	M	3	40.9	45	pT2 Nx	Lower Rectum	CRT + CapOx	IMDA *LVI*	17	No

**(A): Patients underwent TNT after the initial diagnosis. (B): Patients underwent TNT after the transanal full-thickness local excision.**

WW: Watchful Waiting; CCI: Charlson Comorbidity Index; BMI: Body Mass Index; IA: Invasive Adenocarcinoma; IMDA: Invasive Moderately Differentiate Adenocarcinoma; LR: Local Recurrence; DM: Distant Metastasis; LVI: Lymphovascular invasion; DFS: Disease Free Survival after Local Excision (month)

Five patients underwent TNT after transanal full-thickness local excision. No local recurrence or distant metastasis was observed in this group of patients with local excision followed by TNT, with a median follow up of 20 months (IQR 12).

The overall complete response rate following TNT is 93.7%, which consist of both clinical and pathological complete response. Organ preservation rate of the study was 87.5% (Table 1). All patients completed the entire TNT regimen without any toxicity.

## Discussion

Our findings suggest that TNT may be a viable organ-preserving alternative for select patients with early-stage rectal cancer, particularly for low-lying tumors where conventional surgery often results in a permanent stoma.

Traditionally, LARC has been managed with nCRT followed by TME and adjuvant chemotherapy. However, TNT has emerged as a new standard of care [1–3, 11]. Consequently, The National Comprehensive Cancer Network (NCCN) guidelines [1–11] have endorsed TNT for both circumferential resection margin (CRM)-threatening and CRM-negative tumors. In contrast, early-stage rectal cancer (ESRC) management remains primarily surgery-based, with NCCN guidelines recommending transabdominal resection for high-risk cT1 or cT2 tumors [11]. In cases where patients are non-surgical candidates with non-metastatic tumors (cT1 and cN0), ESD is recommended as a local excision technique. However, neoadjuvant therapy is now considered in select cases, particularly when an abdominoperineal resection (APR) would otherwise be required [11].

The outcomes of patients with stage I rectal cancer who underwent TNT have not yet been explored. The GRECCA-12 trial (ClinicalTrials.gov identifier, NCT025114278) is currently in progress, aiming to enhance the percentage of patients with cT2-3 and/or N0 + rectal cancers who are treated using organ-preserving methods by improving tumor response [18]. The study plans to evaluate the effectiveness of induction chemotherapy (comprising 4 cycles of FOLFIRINOX) combined with capecitabine (1600 mg/m2) and radiotherapy (50 Gy, 25 fractions) against chemotherapy alone (capecitabine and 50 Gy radiation), followed by either local excision or radical TME based on the tumor’s response. However, nCRT has been investigated in this setting. Lezoche, et al. [19] found that nCRT plus local excision (LE) resulted in comparable local recurrence rates to TME for select T2N0 rectal cancers, suggesting its feasibility as an organ-preserving strategy. Suzuki et al. [20] and Lee et al. [21] reported acceptable oncologic outcomes with nCRT and LE in T2N0 disease. Additionally, Garcia-Aguilar et al. [22] demonstrated high rates of pathologic complete response (pCR) with nCRT followed by LE. These studies highlight the potential for non-surgical management in carefully selected patients.

While TNT offers advantages such as improved chemotherapy compliance, early micrometastatic control, and higher complete response rates, its toxicity profile must be carefully considered. Prior studies in LARC have reported increased rates of grade 3–4 toxicities with TNT [23–25], though survival outcomes appear unaffected. Data on toxicity in stage I rectal cancer are limited. The ACOSOG Z6041 trial found that reducing CRT doses lowered toxicity rates, underscoring the importance of treatment optimization [22]. Marks et al. [26] reported that CRT before LE increases the rate of wound-related complications (26%) compared to LE alone (0%). However, most complications in their series were classified as minor (82%), and a significant percentage (91%) were managed without additional intervention. While optimizing strategies to achieve a pathologic complete response and favorable oncologic outcomes, it is crucial to avoid overtreatment. The occurrence of chemoradiotherapy-related toxicities underscores the importance of carefully calibrating chemotherapy and radiotherapy doses and effectively managing treatment-related adverse events. Additionally, the consideration of reduced-dose TNT may hold promise in mitigating chemotherapy and radiotherapy-related toxicity in stage I rectal cancer patients.

In subset of 5 patients, local excision was initially performed; however, histopathological assessment revealed either T2 tumors, positive surgical margins, or high-risk features such as perineural invasion or lymphovascular invasion. These findings demonstrated that local excision alone was insufficient as definitive treatment. According to current guidelines, the appropriate curative-intent procedure for such patients is total mesorectal excision. Consequently, the subsequent administration of chemoradiotherapy in this context can still be considered part of a neoadjuvant strategy preceding definitive surgery. We recognize, however, that this the potential for confusion, as the sequence of treatment in these cases does not fit neatly within standard definitions. This highlights the need for more precise terminology in the literature to describe patients who undergo local excision with adverse pathological findings and subsequently require multimodality therapy. In our view, these patients remain candidates for TME—particularly if they experience local recurrence or achieve only a partial response following systemic therapy and radiation.

Efforts persist in prioritizing the avoidance of permanent colostomy and the preservation of organs in patients with early-stage rectal cancer [22]. ESD following TNT demonstrates potential for selected patients with early-stage rectal cancer, potentially improving both functional and oncological outcomes [27]. Additionally, a considerable proportion of patients with stage I rectal cancer undergo APR, resulting in a permanent colostomy. Given the quality-of-life implications for these patients—including the challenges associated with a permanent colostomy and the sexual and voiding dysfunctions linked to conventional surgical methods—broadening the application of TNT in this patient cohort may offer advantages [28]. Notably, in one of our cases, the primary tumor was located adjacent to the internal anal sphincter, raising the possibility of requiring an intersphincteric resection or APR. The fact that this patient ultimately underwent a sphincter-preserving low anterior resection highlights an important consideration for future patient selection.

Furthermore, patients with low-performance status have historically received limited treatment [29]. Compared to postoperative radiotherapy alone, TNT offers the potential advantage of delivering systemic therapy upfront, which may address micrometastatic disease while also reducing the size of the primary tumor prior to definitive surgery. TNT can serve as a viable alternative treatment option for select cases with early-stage distal rectal cancers, either as an initial approach or following local excision in patients who decline or are unfit for surgery [30, 31]. However, it is acknowledged that in frail or high-risk patients, less intensive strategies like reduced dose TNT or radiation alone may be more appropriate, and treatment decisions should be individualized based on patient performance status, comorbidities, and preferences. Nonetheless, comparable results between TNT and radiotherapy alone in early-stage rectal cancer patients as an initial approach or after local excision are not yet available in the current literature, necessitating further investigation.

Despite promising advancements in curative-intent strategies for rectal cancer, several limitations and uncertainties persist. While WW protocols are well-established for LARC patients achieving a clinical complete response after neoadjuvant therapy, their applicability to early-stage rectal cancer remains unclear. Additionally, some rectal cancers exhibit poor response to neoadjuvant treatment for reasons not yet fully understood. The delay in surgical intervention associated with TNT may also be a concern for refractory cases where surgery remains the only definitive treatment. This study is limited by a small sample size, short follow-up duration, and its single-center nature, precluding a comparative analysis. In addition, cohort heterogeneity represents another important limitation, and the terminology remains somewhat ambiguous, as the treatment sequence in patients who underwent local excision does not align neatly with standard definitions. Therefore, these findings should be interpreted with caution, as TNT is not currently a standard of care for stage I rectal cancer. Nonetheless, this study contributes valuable insight into the selective use of TNT in this patient population, highlighting the need for further research to refine treatment strategies.

## Conclusion

While our findings suggest that selective utilization of TNT in stage I rectal cancer may facilitate organ preservation, particularly in low rectal tumors, the results should be interpreted with caution. Reduced-dose TNT may be considered a potential treatment option for early-stage rectal cancer patients in the future. Larger prospective trials with various treatment protocols and extended follow-up periods are essential to validate the efficacy of TNT in patients with stage I rectal cancer.

## Data Availability

Data is available upon request.
